# Incoherent Optical Fluctuation Flowmetry: A New Method for the Assessment of Foot Perfusion in Patients with Diabetes-Related Lower-Extremity Complications

**DOI:** 10.3390/diagnostics12122922

**Published:** 2022-11-23

**Authors:** Polina Glazkova, Alexey Glazkov, Dmitry Kulikov, Sergei Zagarov, Yulia Kovaleva, Alina Babenko, Yulia Kononova, Elena Kitaeva, Timur Britvin, Natalia Mazur, Roman Larkov, Dmitry Rogatkin

**Affiliations:** 1 Moscow Regional Research and Clinical Institute (“MONIKI”), 129110 Moscow, Russia; 2 Medical Faculty, Moscow Region State University, 141014 Mytishchi, Russia; 3 N.A. Semashko National Research Institute of Public Health, 105064 Moscow, Russia; 4 Almazov National Medical Research Centre, 197341 St. Petersburg, Russia

**Keywords:** microcirculation, peripheral artery disease, diabetes mellitus, critical limb ischemia, laser doppler flowmetry, incoherent optical fluctuation flowmetry (IOFF)

## Abstract

(1) Background: To date, there are no studies evaluating the ability of the incoherent optical fluctuation flowmetry (IOFF) method to assess foot tissue perfusion. The aim of this study was to evaluate the correlation between perfusion values measured by IOFF and TcPO_2_ in patients with diabetes-related lower-extremity complications. (2) Methods: This was an observational, cross-sectional, two-center study. Diabetic patients with peripheral artery disease and/or diabetic foot ulcers were studied (n = 27, examinations were carried out on 54 legs). Perfusion in the foot tissues was assessed using TcPO_2_ (reference standard for this study) and the IOFF method. (3) Results: High correlation coefficients of all perfusion parameters measured by IOFF with TcPO_2_ (Rs 0.7 to 0.76) were shown. The study demonstrated that the IOFF method allows, with a sensitivity of 85.7% and a specificity of 90.0%, the identification of patients with a critical decrease in TcPO_2_ < 20 mmHg. (4) Conclusions: The high correlation of IOFF parameters with TcPO_2_ and the moderately high sensitivity and specificity in detecting patients with severe ischemia of foot tissues shows the promise of the method for assessing a tissue perfusion in patients with diabetes-related lower-extremity complications.

## 1. Introduction

Diabetic foot syndrome (DFS) is a complication of diabetes mellitus and is described as a group of symptoms including neuropathy, reduced blood supply and infection leading to tissue breakdown, and morbidity that may be followed by amputation. DFS is one of the most serious complications of diabetes mellitus (DM) [1,2]. Peripheral arterial disease (PAD) often has a more aggressive course in diabetic patients and, in turn, can lead to DFS [3,4]. In the population of people living with DM, PAD is characterized by multilevel atherosclerotic lesions as well as greater involvement of the arteries below the knee [5]

Reliable assessment of foot tissue perfusion in patients with limb ischemia is essential for predicting limb outcomes and choosing the treatment algorithm [6]. Measurements of transcutaneous oxygen tension (TcPO_2_) are widely applied for indirect assessment of foot tissue perfusion in patients with limb ischemia [7]. In a number of studies, TcPO_2_ measurement has shown predictive value in the management of patients with limb ischemia and diabetic foot ulcers [6,8,9]. This method was included in the Wound, Ischemia, and Infection of the Foot (WIfI) classification system, which is recommended for assessing the risk of limb amputation and potential benefit from successful revascularization in patients with lower extremity atherosclerotic occlusive disease [10]. Thus, the results of TcPO_2_ assessment can directly influence the management of patients. The significance of this method (TcPO_2_) increases in patients with DM, as the ankle–brachial index (ABI) may be falsely elevated due to the medial artery calcification [9,11].

However, in addition to a number of clinical limitations (high variability, a dependence on ambient temperature, the presence of edema, a small area of the analyzed tissue, etc.), TcPO_2_ measurement is also limited by the cost of equipment, consumables, and duration of the measurement [6,12,13]. Alternative methods of assessing foot tissue perfusion have either not found clinical application (laser Doppler flowmetry, laser Doppler imaging) or also have a number of limitations (skin perfusion pressure, 2D perfusion imaging, fluorescence angiography, etc.) [6,9,14].

Thus, the development of new tools for the assessment of tissue perfusion in patients with atherosclerotic peripheral artery disease and DFS is an extremely important task.

An incoherent optical fluctuation flowmetry (IOFF) method has been developed in the Laboratory of Medical and Physical Research, Moscow Regional Research and Clinical Institute (“MONIKI”) [15]. This is the first study to assess the clinical perspectives of the method in healthcare.

This study aimed to evaluate the correlation between perfusion values measured by IOFF and TcPO_2_ values in patients with diabetes-related lower-extremity complications.

## 2. Materials and Methods

### 2.1. Study Design, Patients, and Data Sources

This was an observational, cross-sectional, two-center study. The study was conducted at 2 centers:

A: Moscow Regional Research and Clinical Institute (“MONIKI”).

B: Federal State Budgetary Institution “V.A. Almazov National Medical Research Center” of the Ministry of Health of the Russian Federation.

Patients with DM and PAD of lower extremities and/or diabetic foot ulcers were included. PAD was diagnosed by detecting hemodynamically significant stenoses/occlusions of the main arteries of lower extremities. Hemodynamically significant stenoses were defined as the presence of stenosis of at least 50% of the diameter reduction by ultrasound duplex scanning [16,17,18].

Exclusion criteria: pregnancy; diagnosed systemic autoimmune diseases; severe heart rhythm disorders (atrial fibrillation, frequent extrasystoles); presence of acute respiratory viral infections, fever of any genesis; exacerbation of concomitant chronic diseases; blood diseases—thrombocytopenia, anemia (hemoglobin less than 90 g/L); skin diseases that prevent the study; stage 5 chronic kidney disease (glomerular filtration rate < 15 mL/min/1.73 m^2^ according to MDRD or CKD-EPI); taking hormone replacement therapy, oral contraceptives; regular use of steroids, nonsteroidal anti-inflammatory drugs (therapy with antiaggregants was not exclusion criterion).

Macro- and microhemodynamics in the vessels of the lower extremities were assessed in all patients. To verify the diagnosis and assess the macrohemodynamic status in the extremities, all patients underwent ultrasound duplex scanning and ABI measurements.

Perfusion in the foot tissues was assessed both using TcPO_2_ (as the reference standard for this study) and the IOFF method.

**Figure 1:**
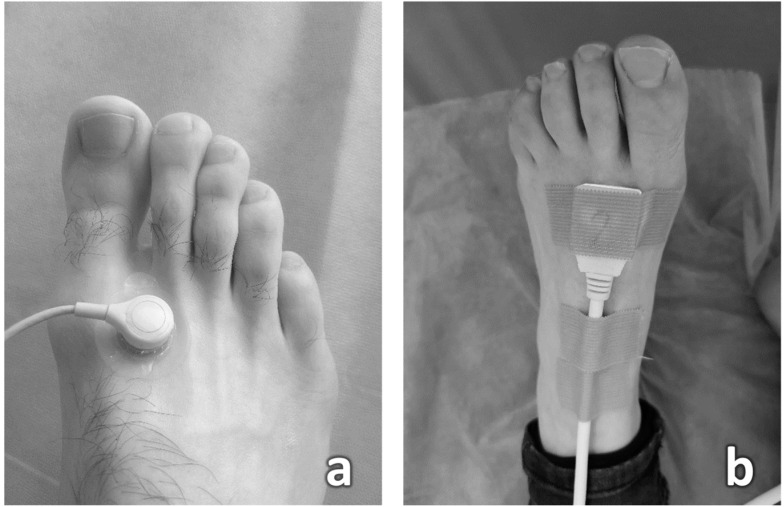
(**a**) TcpO_2_ measurement procedure; (**b**) procedure for measuring perfusion using the IOFF method.

TcPO_2_ was measured by using the TCM4 (Radiometer, Copenhagen, Denmark). The skin was first wiped with an alcohol solution. The sticky fixation ring was fixed at the dorsum of the foot between the first and second metatarsal heads just proximal to the first and second toes. After a few droplets of an electrolyte solution enhancing contact between the electrode and the skin, the electrode was fixed in the fixation ring (Figure 1a). Registration of TcPO_2_ was carried out when they stabilized after 15–20 min of local heating. A probe temperature of 44 °C was selected. The two feet were measured in sequence.

### 2.2. Perfusion Measurement Using the IOFF Method

The foot tissue perfusion measurement using the IOFF method was performed using prototypes of a new diagnostic device developed by the joint-stock company “Elatma Instrument-Making Enterprise” (Ryazan, Russia).

The IOFF method is based on the analysis of low-frequency fluctuations (0–10 Hz) of optical signals backscattered by tissues, initially emitted by the incoherent source—a light-emitting diode (LED). The prototype device uses three LED emission sources operating in the wavelength range of 560–580 nm and one silicon photodiode in the optical sensor. The perfusion value calculated during signal processing is similar to that in laser Doppler flowmetry (LDF) [15,19]. The method allows the assessment of perfusion measured in perfusion units (PU).

The sensor was placed on the dorsal surface of the foot at the first intermetatarsal space (the same location as for the TcPO_2_ electrode) (Figure 1b). The measurement was carried out sequentially, first on the left leg, then on the right one. A total of 54 measurements were taken in 27 patients.

**Figure 2:**
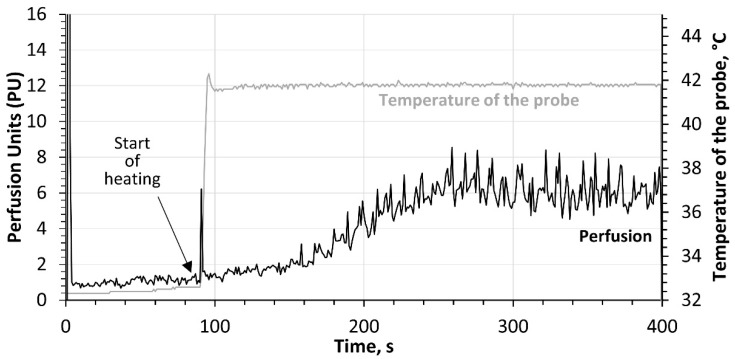
Example of a perfusion curve obtained by IOFF during the local heating test.

Perfusion was recorded during a local heating test. The time duration of the test was 6 min. Using heating plates integrated into the perfusion sensor, a thermoneutral temperature of 32 ± 0.5 °C was maintained for the first 60 s, and the baseline perfusion level (BP) was assessed. The heating plates were then heated at a rate of 1.5 °C/s to a temperature of 42 ± 0.5 °C. This plate temperature was maintained until the end of the measurement (for 5 min). An example of the perfusion curve obtained during the heating test is shown in Figure 2.

The following parameters were assessed:

Baseline perfusion (BP) was calculated as the median baseline perfusion level for the first 60 s of measurement.

Local thermal hyperemia 1–5 min (LTH 1–5 min) was calculated as the median perfusion for each minute of heating.

Before the IOFF and TcPO_2_ measurements, all patients were at rest for 10 min in the supine position, relaxed, head and heels supported, in a room with a comfortable temperature. All patients were asked to refrain from smoking within 3 h prior to the examinations. Thus, the IOFF perfusion measurement and the TcPO_2_ measurement were performed at the same location and under the same conditions (patient positioning and preparation). Because the IOFF perfusion measurement and the TcPO_2_ measurement are accompanied by a heat test, measurements were taken on different days.

### 2.3. Assessment of Macrohemodynamic Parameters

The ABI of each foot was calculated by dividing the higher pressure in the posterior tibial or dorsalis pedis arteries by the higher systolic blood pressure in the right or left arm. To record blood pressure, a Doppler probe was placed over the pulsing artery at a 45° to 60° angle to the surface of the skin [20].

The level and severity of arterial stenosis were assessed by the duplex ultrasound scanning of the lower limb arteries using the Philips Affinity 50 (Philips Ultrasound, Tampa, Florida, USA) and the Vivid 7 Dimension (GE Healthcare, Chicago, Illinois, USA). The ultrasound protocol involved assessing the presence of hemodynamically significant stenoses (at least 50% of the diameter reduction) in 6 main arteries of each lower limb (common femoral artery, deep femoral artery, superficial femoral artery, popliteal artery, anterior tibial artery, posterior tibial artery).

### 2.4. Statistical Analysis

Statistical analysis of the data was carried out using the RStudio 2021.09.0 Build 351 program using the R version 4.1.1 language. Medians and quartiles (Me (LQ; UQ)) were calculated for quantitative variables. Absolute (n) and relative (%) frequencies were used for qualitative variables. Spearman’s correlation coefficient was used to assess the correlation between quantitative variables. Thresholds for quantitative variables were estimated using ROC analysis (pROC 1.18.0 package). The required sample size was calculated using the “power.roc.test()” function. The level of the type I error (α) was set equal to 0.05; null hypotheses were rejected at *p* < 0.05.

The required sample size was calculated using the power.roc.test() function from the pROC package. The power of the study was set at 90%. The expected area under the ROC curve for identifying limbs with low TcPO_2_ (<20 mmHg) was 0.8. The ratio of the number of limbs without TcPO_2_ reduction to the number of limbs with TcPO_2_ reduction was set at 2 to 1 (κ = 2). Thus, at least 39 observations had to be recruited into the study in order to achieve a 90% power level under these conditions. Since the different limbs of the patients were analyzed as independent cases, at least 20 patients had to be included in the study.

## 3. Results

### 3.1. Study Population and Baseline Characteristics

A total of 27 patients were included in the study. The study was carried out on 54 feet. The characteristics of the group are listed in Table 1.

### 3.2. Comparison of IOFF and TcPO_2_ Measurement Results

It was revealed that all perfusion parameters analyzed by the IOFF method correlated significantly with the TcPO_2_ measurement with high correlation coefficients (Table 2).

In order to assess this phenomenon in detail, all measurements were divided into three subgroups: subgroup 1—TcPO_2_ < 20 mmHg (n = 14); subgroup 2—TcPO_2_ 20–39 mmHg (n = 17); subgroup 3—TcPO_2_ ≥ 40 mmHg (n = 23). These TcPO_2_ limits have been used because, according to a number of studies, ulcer healing and limb prognosis are generally poor if TcPO_2_ is <20 mmHg and are generally good if >40 mmHg [7].

**Figure 3:**
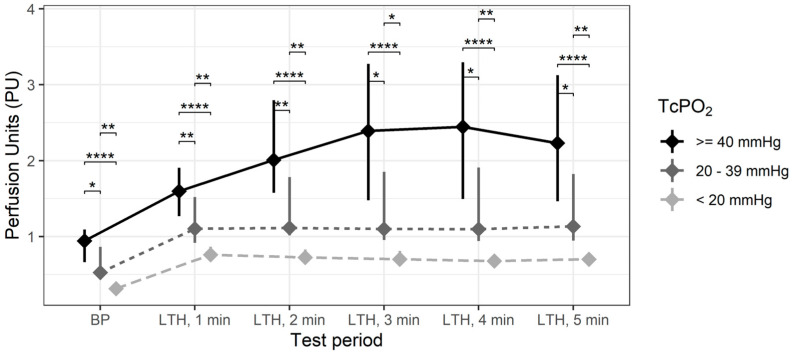
Median and interquartile ranges of perfusion measured by IOFF at different time points of the local heating test in legs with different TcPO_2_ levels (*—*p* < 0.05, **—*p* < 0.01, ****—*p* < 0.0001).

The perfusion parameters estimated by the IOFF method were analyzed in each of the subgroups separately and then compared (Figure 3).

Baseline perfusion and local thermal hyperemia measured by the IOFF method differed significantly in all three subgroups. Limbs with critically low transcutaneous oxygen tension (TcPO_2_ < 20 mmHg) had a significantly lower baseline perfusion than those in the subgroups 2 and 3. In the first subgroup, the increase in perfusion in response to heating was also significantly less pronounced than in the limbs with higher TcPO_2_ values. In this subgroup, there was low variability in all analyzed perfusion parameters. In cases where the TcPO_2_ value was greater than 40 mmHg, the levels of BP and LTH were significantly higher than in the subgroups 1 and 2. The hyperemic response peaked at 3 and 4 min of heating.

Receiver operating characteristic (ROC) curve analyses were performed to assess the ability to identify patients with a critical decrease in TcPO_2_ (<20 mmHg) based on the IOFF perfusion measurements (Table 3).

All analyzed perfusion parameters from the IOFF measurement had a high diagnostic potential in identifying patients with critically low TcPO_2_. The area under the ROC curve for all IOFF parameters, including BP, was higher than that for ABI.

**Figure 4:**
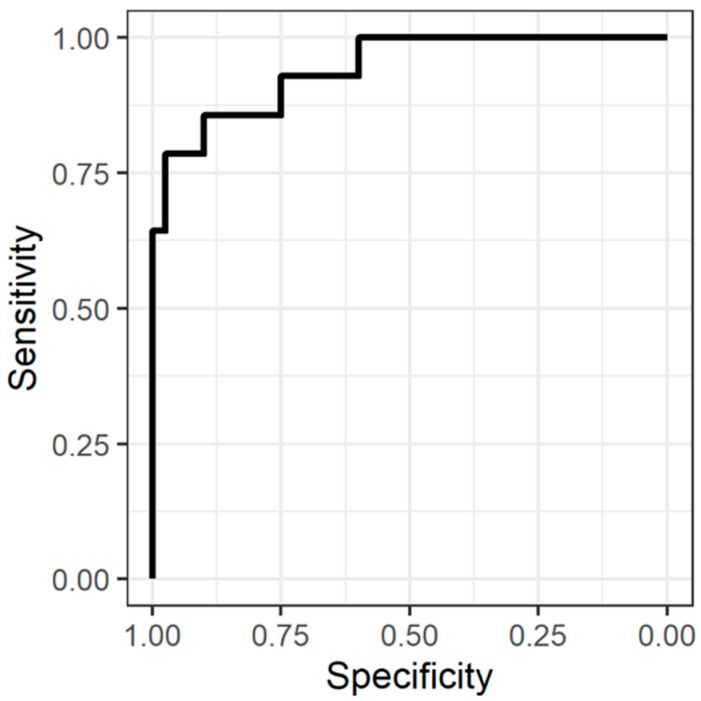
ROC curve showing the sensitivity and specificity of LTH, 2 min for identification of legs with a critical decrease in TcPO_2_ (<20 mmHg).

The area under the curve (AUC) of the LTH, 2 min was 0.943 (95% CI 0.874–1) and had an optimal cutoff value for the identification of limbs with critically low TcPO_2_ (0.989 PU), with a sensitivity of 85.7% and a specificity of 90% according to the ROC analysis (Figure 4).

## 4. Discussion

This study was the first to investigate the informative value of the IOFF technique in assessing a foot tissue perfusion in patients with diabetes-related lower-extremity complications. High correlation coefficients of all perfusion parameters measured by IOFF with TcPO_2_ (Rs 0.7 to 0.76) were demonstrated. The study showed that the IOFF method allows, with a moderately high sensitivity of 85.7% and a specificity of 90.0%, the identification of patients with a critical decrease in TcPO_2_ < 20 mmHg. Such a decrease in TcPO_2_ is known to indicate a poor prognosis for ulcer healing and limb preservation, so identifying patients in this group is of a great clinical importance [7,21].

Current assessment standards in lower extremity artery disease focus on macrovascular function with less emphasis on foot tissue perfusion measurements. However, measurement of foot perfusion is extremely important because macro- and microvascular disorders are not always congruent [22]. A variety of noninvasive diagnostic technologies have been proposed as promising methods for assessing foot tissue perfusion (LDF, LDI, Laser speckle contrast imaging, 2D perfusion imaging, fluorescence angiography, near-infrared spectroscopy, cone-beam computed tomography, etc.) [6,22,23,24,25,26]. However, most of these methods have not yet become widespread in clinical practice [6,25].

The ideal method for the assessment of a foot tissue perfusion should be inexpensive, readily available, and reproducible, and its results should help the clinician to predict wound healing and provide information that influences patient management [6]. The development of such new methods is complicated by the lack of a widely applicable, highly sensitive method that could be called the “gold standard” for studying perfusion and microcirculation in tissues. In this study, TcPO_2_ measurement was used as a reference method. TcPO_2_ method has been shown to be highly informative in predicting the ulcer healing and foot amputation, but the method is limited in application due to the cost of equipment, consumables, and duration of the measurements [6,12,13]. Thus, the development of new, widely available methods of assessing foot tissue perfusion can significantly improve the quality of management of patients with diabetes-related lower-extremity complications.

A number of studies have evaluated the applicability of LDF and laser Doppler imaging as a noninvasive optical method for assessing a tissue perfusion [6,27,28,29,30]. However, these tools have not found widespread clinical application due to the high frequency of artefacts, sensitivity to ambient temperature, poor reproducibility, and high operator dependence. In addition, the disadvantage of the LDF method is the very small volume of probed tissue (1 mm^3^ or smaller) [9,31]. Previously, LDF has been shown to be less predictive of ulcer healing and forefoot amputation than the TcPO_2_ testing [28].

The new IOFF method makes it possible to analyze the perfusion index similar to that calculated by the LDF method. The use of LEDs makes it possible to significantly reduce the cost of the sensors and device. LEDs also eliminate the need for lasers and fiber optics. This reduces the impact of wire positioning on instrument performance. The penetration depth at IOFF is 2–3 mm, which is slightly greater than in LDF. In addition, because of the probe design, the signal is analyzed from a larger area of skin (~25 mm^2^). Due to this, the signal backscattered from the tissue is collected from a larger tissue volume than in LDF and includes deeper vascular plexuses and larger vessels [14,15]. This also reduces the impact of local vascular network heterogeneity on measurement variability.

The assessment of perfusion by IOFF and TcPO_2_ are similar. Both procedures involve the local heating test, and the measurement results are affected by a tissue blood supply. However, perfusion assessment using the IOFF method is significantly less time consuming than measuring TcPO_2_ (6 min vs. 20 min). In addition, the IOFF method does not require expensive consumables, and the use of the light-emitting diodes makes the technology easy to implement and potentially widely available. Expected cost of IOFF devices would be about USD 2000. This low estimated cost is an important potential advantage of the IOFF method over other methods of perfusion assessment (such as LDF, TcPO_2,_ 2D perfusion imaging, cone-beam computed tomography, etc.)

Thus, the new method IOFF may be promising as an informative, available and convenient way to assess foot tissue perfusion in patients with diabetes-related lower-extremity complications.

Additionally in this study, the IOFF method was compared with the results of measuring ABI. The area under the ROC curve for all IOFF parameters was higher than that for ABI for identification legs with a critical ischemia. However, it is known that the usefulness of the ABI is limited in people with DM because of medial arterial calcification [11]. Thus, in this population, the toe–brachial index (TBI) may be more informative [32]. However, this index is not applicable for patients with an amputated big toe. Though TBI was not measured in our study, it will be of great interest to compare the results of TBI and IOFF assessment in further studies.

This first study of the IOFF technique foreshadows further necessary longitudinal research, which should focus on endpoint analysis and the derivation of specific perfusion thresholds for the probability of wound healing. For a more accurate assessment of the applicability of the method IOFF in practical healthcare, further study of reproducibility, testing of the methodology on large sample sizes, and evaluation of the predictive ability of the method is required. It is also of great interest to explore the association between flow and perfusion [33] and to assess the diagnostic informativeness of the method as a screening tool for the detection of PAD in both diabetic and nondiabetic patients.

## 5. Limitations

In this study, we used strict inclusion/exclusion criteria. This reduced possible measurement bias (e.g., associated with heart rhythm disorders or anemia) but may affect the reproducibility of the result in “real-world practice”.

It is known that the duplex ultrasound scanning is an operator-dependent procedure. The study was conducted at two independent centers, so the assessment of the duplex ultrasound scanning was performed by several experts, which could affect the accuracy.

## 6. Conclusions

The results of the pilot study demonstrated a high correlation between the perfusion parameters assessed by IOFF and TcPO_2_. A sensitivity of 85.7% and specificity of 90.0% in identifying patients with critically decreased TcPO_2_ < 20 mmHg suggests that the IOFF technique may be promising as an informative, rapid, and noninvasive method of assessing tissue perfusion.

## Figures and Tables

**Table 1:** Characteristics of the studied patients.

Parameter	Value
Sex: male/female, n (%)	19 (70.4%)/8 (29.6%)
Age, years, Me (LQ; UQ)	64 (56; 68)
Body mass index, kg/m^2^, Me (LQ; UQ)	28.4 (26.5; 33.9)
Legs with hemodynamically significant artery stenoses by duplex ultrasound	39 (72.2%)
Feet with lower-extremity ulcers, n	14 (51.9%)
Neuropathic foot ulcers	7 (50%)
Ischemic foot ulcers	2 (14.3%)
Neuro-ischemic foot ulcers	5 (35.7%)
ABI (recorded separately for each limb), Me (LQ; UQ)	0.94 (0.88; 0.98)
>1.4, n (%)	1 (1.8%)
1–1.4, n (%)	7 (13%)
0.91–0.99, n (%)	18 (33.3%)
0.4–0.9, n (%)	16 (29.6%)
0.4, n (%)	1 (1.8%)
not defined, n (%) *	11 (20.3%)
TcPO_2_ (recorded separately for each limb), Me (LQ; UQ)	34 (18; 48)
TcPO_2_ < 20 mmHg;	14 (25.9%)
20–39 mmHg;	17 (31.5%)
TcPO_2_ ≥ 40 mmHg.	23 (42.6%)
MNSI (Part A), Me (LQ; UQ)	9 (7; 9)
MNSI (Part B), Me (LQ; UQ)	7.00 (5.00; 7.75)
HbA1c, %, Me (LQ; UQ)	8.1 (7.12; 9.45)
eGFR according to CKD-EPI (mL/min/1.73 m^2^), Me (LQ; UQ)	82 (67; 86)
**Comorbidities**	
Hypertension, n (%)	25 (92.6%)
Chronic heart failure, n (%)	10 (37%)
Angina pectoris, n (%)	10 (37%)

ABI—ankle-brachial index; eGFR using CKD-EPI—Estimated Glomerular Filtration Rate according to Chronic Kidney Disease Epidemiology Collaboration; HbA1—glycated hemoglobin; LQ—lower quartile; Me—median; UQ—upper quartile; MNSI—The Michigan Neuropathy Screening Instrument. * ABI was not detected on 11 limbs due to the absence of a pulse in the arteries of the foot or due to severe pain syndrome.

**Table 2:** Correlation between the parameters of the IOFF signal and value of the TcPO_2_ assessed on 54 measurements (27 patients). The table shows the correlation coefficients.

	TcPO_2_	*p*
BP	0.70	<0.001
LTH, 1 min	0.74	<0.001
LTH, 2 min	0.76	<0.001
LTH, 3 min	0.73	<0.001
LTH, 4 min	0.75	<0.001
LTH, 5 min	0.74	<0.001

BP—baseline perfusion; LTH 1–5 min—local thermal hyperemia for each minute of heating; *p*—statistical significance.

**Table 3:** Area under the ROC curve showing the diagnostic potential of IOFF and ABI for identification legs with a critical decrease in TcPO_2_ (<20 mmHg).

	AUC	LCL	UCL
ABI	0.881	0.689	1
BP	0.927	0.831	1
LTH, 1 min	0.939	0.863	1
LTH, 2 min	0.943	0.874	1
LTH, 3 min	0.914	0.802	1
LTH, 4 min	0.941	0.865	1
LTH, 5 min	0.927	0.832	1

ABI—ankle-brachial index; AUC—area under the ROC curve; LCL—lower 95% confidence limit; UCL—upper 95% confidence limit; LTH 1–5 min—local thermal hyperemia for each minute of heating; BP—baseline perfusion.

## Data Availability

The data that support the findings of this study are available from the corresponding author (Glazkova P.) upon reasonable request.
